# *CIS* deletion by CRISPR/Cas9 enhances human primary natural killer cell functions against allogeneic glioblastoma

**DOI:** 10.1186/s13046-023-02770-6

**Published:** 2023-08-10

**Authors:** Tsutomu Nakazawa, Takayuki Morimoto, Ryosuke Maeoka, Ryosuke Matsuda, Mitsutoshi Nakamura, Fumihiko Nishimura, Noriko Ouji, Shuichi Yamada, Ichiro Nakagawa, Young Soo Park, Toshihiro Ito, Hiroyuki Nakase, Takahiro Tsujimura

**Affiliations:** 1 Grandsoul Research Institute for Immunology, Inc, 8-1 Matsui, Uda, Nara 634-8522 Japan; 2 Clinic Grandsoul Nara, Uda, Nara, Japan; 3 Department of Neurosurgery, Nara Medical University, Kashihara, Nara, Japan; 4 Department of Immunology, Nara Medical University, Kashihara, Nara 634-8522 Japan

**Keywords:** Natural killer cell, Glioblastoma, CRISPR/Cas9, CIS, Peripheral blood

## Abstract

**Background:**

Glioblastoma (GBM) is the most common malignant brain tumor and has “immunologically cold” features. Changing GBM to an “immunologically hot” tumor requires a strong trigger that induces initial immune responses in GBM. Allogeneic natural killer cells (NKCs) have gained considerable attention as promising immunotherapeutic tools against cancer, where gene-edited NKCs would result in effective anti-cancer treatment. The present study focused on the immune checkpoint molecule cytokine-inducible SH2-containing protein (CISH, or CIS) as a critical negative regulator in NKCs.

**Methods:**

The GBM tumor environment featured with immunological aspect was analyzed with Cancer immunogram and GlioVis. We generated human primary CIS-deleted NKCs (NK dCIS) using clustered regularly interspaced short palindromic repeats/CRISPR-associated protein 9 (CRISPR/Cas9) with single guide RNA targeting genome sites on CIS coding exons. The genome-edited NKCs underwent microarray with differential expression analysis and gene set enrichment analysis (GSEA). The anti-GBM activity of the genome-edited NKCs was evaluated by apoptosis induction effects against allogeneic GBM cells and spheroids. We further detected in vivo antitumor effects using xenograft brain tumor mice.

**Results:**

We successfully induced human CIS-deleted NKCs (NK dCIS) by combining our specific human NKC expansion method available for clinical application and genome editing technology. *CIS* gene-specific guide RNA/Cas9 protein complex suppressed CIS expression in the expanded NKCs with high expansion efficacy. Comprehensive gene expression analysis demonstrated increased expression of 265 genes and decreased expression of 86 genes in the NK dCIS. Gene set enrichment analysis revealed that the enriched genes were involved in NKC effector functions. Functional analysis revealed that the NK dCIS had increased interferon (IFN)ɤ and tumor necrosis factor (TNF) production. *CIS* deletion enhanced NKC-mediated apoptosis induction against allogeneic GBM cells and spheroids. Intracranial administration of the allogeneic NKCs prolonged the overall survival of xenograft brain tumor mice. Furthermore, the NK dCIS extended the overall survival of the mice.

**Conclusion:**

The findings demonstrated the successful induction of human primary NK dCIS with CRISPR/Cas9 with efficient expansion. *CIS* deletion enhanced the NKC-mediated anti-tumor effects in allogeneic GBM and could be a promising immunotherapeutic alternative for patients with GBM.

**Supplementary Information:**

The online version contains supplementary material available at 10.1186/s13046-023-02770-6.

## Introduction

The most frequent and aggressive brain tumor is glioblastoma (GBM), which was categorized as a grade IV astrocytoma (highest-grade and most malignant glioma) in the 2021 World Health Organization ordering of central nervous system tumors [1]. To date, the prevailing GBM treatment has been maximal safe resection; subsequently, adjuvant radiotherapy and temozolomide (a representative DNA-alkylating agent for glioma) chemotherapy are administered. Unfortunately, the median overall survival (mOS) of 15–17 months remains low. Furthermore, the rate of 5-year overall relative survival is 5.8% [2, 3]. The mOS was significantly improved (20.5 months) by including tumor-treating fields in temozolomide chemotherapy [4]. Recently, a phase II trial of intratumoral oncolytic herpes virus G47Δ for residual or recurrent GBM yielded a good safety profile and favorable survival benefits [5]. Nonetheless, the poor prognosis of patients with GBM warrants more research into novel GBM treatment strategies, which include immunotherapy.

Most immunotherapies primarily aim to obtain a tumor-specific immune response that selectively eliminates cancer cells by activating T cells. T cell-based immunotherapy, e.g., chimeric antigen receptor (CAR) T cell therapies and checkpoint inhibitors, are typical immunotherapies that have been used in other solid tumors and hematologic malignancies [6–8]. Although there have been several studies on GBM immunotherapies, few effective strategies were discovered [9, 10]. Given their high cytotoxicity and T cell receptor/human leucocyte antigen (HLA)-unrestricted effector function, natural killer cells (NKCs) have become an encouraging alternative platform for T cell-based immunotherapy platform [11]. Immunotherapy based on NKCs exemplifies a cancer therapeutic approach that is unlike T cell-based therapies. Unlike T cells, NKCs use multiple activating and inhibitory receptors to recognize tumors that comprise heterogeneous cells even when major histocompatibility complex class I (MHC class I) molecules [human leukocyte antigen class I (HLA class I) in humans] are absent or reduced [12]. Early clinical trials examined ex vivo expanded autologous NKC adoptive transfer as a possible therapy for various cancers. Despite demonstrating low toxicity and eliciting a positive treatment response, the anti-tumor effect was restricted [13–15]. The main barrier in NKC adoptive transfer was functional inhibition due to self-recognition via the inhibitory killer cell immunoglobulin-like receptors (KIRs) present on NKCs, which are equivalent to the presence of HLA class I on cancer cells. This recognition resulted in activation blockade [16]. Contrastingly, allogeneic NKCs have KIR/HLA class I mismatch and can attack cancer cells. Several clinical reports indicated that allogeneic NKCs might provoke regression in hematological malignancies, e.g., multiple myeloma and acute myeloid leukemia (AML) [17, 18]. A phase I trial partial report demonstrated complete remission in five of nine patients with refractory AML, and two patients exhibited significant transient loss of leukemic cells, yielding a 77.8% response rate [19]. This result was comparable to the initial US trial data of CD19-targeted CAR-T cells for treating juvenile acute lymphoblastic leukemia [20]. Differing from CAR-T therapy, these treatments were safe without causing significant toxicity, e.g., neurotoxicity, cytokine release syndrome (CRS), or graft-versus-host disease (GVHD). These findings indicated that allogeneic NKC-based immunotherapy could be promising for GBM.

In view of the strong immunosuppressive GBM tumor microenvironment (TME) [21, 22], further NKC alteration is required to devise a cure for GBM. We hypothesized that clustered regularly interspaced short palindromic repeats (CRISPR) and CRISPR-associated protein 9 (Cas9) targeting of intracellular inhibitory molecule (immune checkpoint molecule) was a means of improving allogeneic NKC-based immunotherapy for GBM, which is comparable to the method of blocking critical immune checkpoints. CRISPR/Cas9 disturbs the target gene via small RNAs that chaperone the Cas9 DNA nuclease to the target site through base pairing; this approach is extremely specific and efficient to engineer and disrupt eukaryotic genomes [23, 24].

The suppressor of cytokine signaling (SOCS) protein (cytokine-inducible SH2-containing protein, CISH, or CIS) regulates NKC effector function negatively via the suppression of interleukin (IL)-2 and IL-15 signaling through the blockade of Janus kinase–signal transducer and transcription activator (JAK–STAT). The loss of CIS caused increased and extended JAK–STAT signaling in NKCs [25]. CRISPR/Cas9 targeting of CIS could release human NKC effector function and result in unparalleled anti-tumor effects against GBM.

In clinical trials, NKCs were induced by deleting the *CIS* gene from umbilical cord blood cells and induced pluripotent stem cells and their anti-tumor effects in several cancers were examined [26, 27]. Nevertheless, these induction methods are not widely available due to the long culture duration and complex culture techniques. An alternative method is to induce *CIS*-deleted NKCs from human peripheral blood. Rautela et al. deleted *CIS* from human primary NKCs to demonstrate that CIS regulated human NKC effector functions [28]. However, another group that performed CRISPR/Cas-mediated *CIS* knockout in primary human NKCs did not report enhanced anti-tumor activity [29]. The effect of deleting *CIS* in primary human NKCs remains contentious and only partly clarified. We consider the efficiency of expansion in both reports low and that it did not reach clinical application level. Furthermore, the detailed and accurate characterization of human primary *CIS*-deleted NKCs (NK dCIS) against GBM has not been described in vitro and in vivo. Previously, we established a unique technique to efficiently expand highly purified NKCs from human peripheral blood [30]. A combination of the NKC expansion approach and CRISPR/Cas9 would enable the obtainment of abundant clinically applicable NK dCIS and facilitate comprehensive and precise characterization against GBM.

In the present study, NK dCIS were induced from human peripheral blood ex vivo with CRISPR/Cas9-based genome editing and their anti-tumor effects in allogeneic GBM were evaluated.

## Materials and methods

### Ethics

This Ethics Committee of Nara Medical University approved this study (No. 1058). The study was conducted according to university guidelines. All procedures that involved human participants were conducted according to institutional and/or national research committee ethical standards and the 1964 Declaration of Helsinki and its subsequent alterations or equivalent ethical standards. All healthy volunteers in this study provided informed consent in accordance with Declaration of Helsinki tenets.

### Cancer immunogram and GlioVis

The cancer immunogram is used for illustrating the condition of several simultaneous parameters that affect the interaction between a cancer and the immune system. The proposed immunogram is a radar plot where each axis acts as a scale to enumerate parameters (immunosuppressive regulators, immune cell infiltration and so on. Our immunogram analysis results of 9417 RNA-seq data from 9362 patients with 29 different solid cancers in The Cancer Genome Atlas (TCGA) dataset were obtained from the RNA sequencing (RNA-seq)-based Cancer Immunogram Web (https://yamashige33.shinyapps.io/immunogram/). Subgroups of patients with each cancer type or the entire TCGA cohort were normalized [31].

Characteristic immune cell marker (CD3, CD20, NKp46, CD11b, CD11c, TMEM) expression patterns in glioma were detected by examining glioma RNA-seq data from TCGA Database in the GlioVis data portal (http://gliovis.bioinfo.cnio.es/) [32].

### Cell lines

We obtained the human GBM cell lines U87MG from American Type Culture Collection (Manassas, VA, USA). We obtained the T98G and U251MG GBM cells from RIKEN BioResource Center (Tsukuba, Japan) and JCRB Cell Bank (Osaka, Japan), respectively. The cell lines were authenticated and tested as mycoplasma-free. We maintained the cells in Dulbecco’s modified Eagle’s medium (DMEM; Life Technologies, Carlsbad, CA, USA) containing 100 µg/ml streptomycin, 100 U/ml penicillin (Thermo Fisher Scientific, Waltham, MA, USA), and 10% heat-inactivated fetal bovine serum (FBS; MP Biomedicals, Tokyo, Japan) at 37 °C in a humidified atmosphere with 5% CO_2_.

### Single guide RNAs

We prepared two single guide RNAs (sgRNAs) targeting the exon 3 or 4 regions of the human *CIS* gene located on 3q13.31 according to the manufacturer’s instructions (IDT, Coralville, IA, USA, https://sg.idtdna.com/site/order/designtool/index/CRISPR_PREDESIGN). We selected targets with high on-target potential values (> 60%) and low off-target risk values (> 80%) as calculated by the IDT algorithm. Subsequently, we selected the targets that did not contain any exon regions in the top 10 rankings of off-target predictions by the IDT algorithm. The CIS exon 3 and 4 target sequences were as follows: CTCACCAGATTCCCGAAGGTTGG and CGTACTAAGAACGTGCCTTCTGG, respectively. The underlined sections indicate the protospacer adjacent motif (PAM) sequence.

The negative control sgRNA was obtained from IDT. The sgRNA sequence is as follows: rCrGrUrUrArArUrCrGrCrGrUrArUrArArUrArCrGrGrUrUrUrUrArGrArGrCrUrArUrGrCrU.

### Induction of NK dCIS

The highly purified human NKCs were expanded as previously described [30]. Peripheral blood mononuclear cells (PBMCs) were obtained from 16 mL heparinized peripheral blood from three healthy male volunteers (51, 47, and 43 years old). The PBMC CD3 fraction was depleted using RosetteSep™ Human CD3 Depletion Cocktail (STEMCELL Technologies, Vancouver, Canada). The CD3-depleted PBMCs (10^7^ cells) were placed for 7 days in a T25 culture flask (Corning, Steuben, NY, USA) that contained 10 mL AIM-V medium (Life Technologies) with supplementation of 50 ng/mL recombinant human IL-18 (rhIL-18, Medical & Biological Laboratories Co., Ltd., Nagoya, Japan), 10% autologous plasma, and 3000 IU/mL rhIL-2 (Novartis, Basel, Switzerland) at 37 °C in a humidified atmosphere with 5% CO_2_. The AIM-V medium containing 3000 IU/mL rhIL-2 was refilled as required.

Genome editing was conducted as previously described with minor modifications [33]. Expanded NKCs (3 × 10^6^) were electroporated to ribonucleoprotein (RNP) complexes [targeted sgRNA/transactivation CRISPR RNA (tracrRNA) and recombinant Cas9 (IDT)] using a Human NK Cell Nucleofector Kit (VPA-1005; Lonza, Basel, Switzerland) and electroporation program X-001. Subsequently, we resuspended the cells in AIM-V medium with 10% autologous plasma and 3000 IU/mL rhIL-2 and placed them for 7 days in a 12-well plate (Corning) at 37 °C in a humidified atmosphere with 5% CO_2_.

Expansion ratios were determined by dividing the cell number at day 3, 5, and 7 post-electroporation by the cell number at day 1 post-electroporation.

### Efficacy of CRISPR/Cas9 gene disruption

We harvested the genome-edited NKCs 7 days after electroporation, extracted their DNA with a QIAamp DNA mini kit (Qiagen, Hilden, Germany) and performed T7 endonuclease 1 (T7E1) mismatch detection assays using an Alt-R Genome Editing Detection Kit (IDT) as described previously [33, 34]. We amplified the on- and off-target (OT and OF, respectively) sites and adjacent sequences from the genomic DNA with KOD FX enzyme solution (TOYOBO, Osaka, Japan). The PCR conditions were as follows: one cycle at 94 °C for 2 min, then 40 cycles at 98 °C for 10 s, 63 °C for 30 s, and 68 °C for 30 s, and a final cycle at 68 °C for 7 min. We performed the PCR on a LifeECO thermal cycler (Bioer Technologies Co. Ltd., Hangzhou, China). The PCR primers were obtained from Thermo Fisher Scientific.

The subsequent PCR was performed on the LifeECO thermal cycler with the following cycling conditions: 95 °C for 5 min, decrease from 95 °C to 85 °C at a rate of 2 °C per second, decrease from 85 °C to 25 °C at a rate of 0.1 °C per second, then decreased to 4 °C. We digested the rehybridized PCR products for 30 min with T7E1 and separated them for 20 min on 2% agarose gel. We visualized the DNA under a UV transilluminator (FAS-IV, Nippon Genetics Co. Ltd., Tokyo, Japan). OF mutagenesis, which was predicted with a gene homology-based off-targeting potential checking system (IDT) was detected in the same manner. The PCR primers used to amplify the target locus are listed in Supplemental Materials and Methods 1.

### Microarray-based gene expression

We examined mock NKCs (NK mock) and NK dCIS gene expression (National Center for Biotechnology Information Gene Expression Omnibus [NCBI GEO] accession no. GSE229085) using the Clariom™ S array and deposited into NCBI GEO. We analyzed all CEL files using Transcriptome Analysis Console (TAC 4.1, Thermo Fisher Scientific). The mRNA signal intensity (log_2_) was corrected by calculating the characteristic control signal values. Gene set enrichment analysis (GSEA) was performed to establish whether a predefined set of genes differed statistically significantly between two biological states. We uploaded the microarray data to the GSEA website (https://www.gsea-msigdb.org/gsea/index.jsp).

#### Antibody staining and flow cytometry

We stained the cells with the appropriate antibodies and fixed them for 1 h in 1% paraformaldehyde-containing phosphate-buffered saline (PBS) at 4 °C. We obtained the data using a BD FACSCalibur flow cytometer (BD Biosciences, San Jose, CA, USA) and analyzed it using FlowJo v10 (BD Biosciences). We determined CD107a expression with an IMMUNOCYTO CD107a Detection Kit (MBL, Nagoya, Japan) according to the instructions.

For intracellular cytokine staining, we stimulated the cells in a 96-well round-bottom plate with T98G GBM cells at 37 °C for 4 h, then incubated them for 5 h at 37 °C with Protein Transport Inhibitor Cocktail (Thermo Fisher Scientific). Then, we fixed and permeabilized the cells with BD Cytofix/Cytoperm Kit (BD Biosciences) and incubated them for 30 min with anti-cytokine antibodies on ice. Phosphorylation was detected with PerFix-EXPOSE (Beckman Coulter, Brea, CA, USA) according to the instructions. The Supplementary Materials and Methods list the antibodies used for the flow cytometry.

### Western blotting

NKCs (10^6^) were dissolved in RIPA Lysis and Extraction Buffer with Halt Protease Inhibitor Cocktail (Thermo Fisher Scientific) and sonicated by the ultrasound-based Sonifier 250 homogenizer (Branson, Hannover, Germany) according to the manufacturer’s instruction. We mixed the whole cell lysate with 4 × Bolt LDS Sample Buffer (Thermo Fisher Scientific) and incubated it for 10 min at 70 °C. Subsequently, we performed 4–12% sodium dodecyl sulfate–polyacrylamide gel electrophoresis using 5 µL (for glyceraldehyde-3-phosphate dehydrogenase [GAPDH]) to 40 µL (for CIS) lysate, then transferred the blots onto a PVDF membrane using the iBlot 2 Dry Blotting System (Thermo Fisher Scientific). We reacted the membranes at room temperature using iBind Automated Western Systems (Thermo Fisher Scientific). The primary antibodies were rabbit polyclonal IgGs against CIS (1:500 dilution, clone D4D9, Cell Signaling, Danvers, MA, USA) and GAPDH (1:1000 dilution, clone 14C10, Cell Signaling). The secondary antibody was horseradish peroxidase-conjugated anti-rabbit IgG antibody (1:200 dilution, Cell Signaling). The blots were developed with SuperSignal West Pico PLUS Chemiluminescent Substrate (Thermo Fisher Scientific). We determined the signal intensity with FUSION Solo and FUSION Solo 7S Edge (Vilber Bio Imaging, Paris, France).

### Growth inhibition assays

We examined the inhibitory effects of the genome-edited NKCs on GBM cells with xCELLigence RTCA (real-time cell analysis) S16 and DP instruments (ACEA Biosciences, San Diego, CA, USA). The RTCA instruments function based on the electronic impedance reading from gold-plated sensor electrodes at the bottom of the adhesion and cytotoxicity plates (E-plate 16; ACEA Biosciences). The electronic readings change as cells attach or detach from the surface electrodes and thereby produce a change in impedance that is calculated via complex mathematical algorithms and plotted as cell index values. The number of attached cells and the cell index value readout on the instrument are directly correlated, and vice versa. The normalized cell index demonstrates that a specific time (in this study, when NKCs were added) is selected and set by the software as 1.0 and all values are depicted as a percentage of this. The cell index value variation for each sample well can be standardized by calculating the normalized cell index [35]. Cytotoxicity-mediated growth inhibitory effects were tested as described previously [33, 34, 36]. Briefly, we added 100 µL complete medium to each well on an E-plate 16 (ACEA Biosciences). We measured the background impedance at 37 °C in a humidified atmosphere with 5% CO_2_. We seeded the GBM cells (2 × 10^4^ T98G, U251MG, U87MG cells/well) as the target (T) cells and recorded the impedance every 5 min for 72 h. After 24 h, we added the genome-edited NKCs to each well as effector (E) cells in E:T cell ratios of 1:1. We analyzed the data using RTCA version 1.2 (ACEA Biosciences) and calculated the relative growth inhibition as follows: (1 − normalized cell index of target cells co-cultured with each sample ÷ normalized cell index of target cells) × 100 (%).

### Spheroid culture and CFSE-based cytotoxic assay

We seeded the GBM cells (300–6000 cells/well) onto nonadherent V-bottom 96-well plates (PrimeSurface 96U, MS-9096 V, Sumitomo Bakelite, Tokyo, Japan) in DMEM supplemented with 10% FBS and cultured them at 37 °C in a humidified atmosphere with 5% CO_2_ for 1 days. For fluorescence microscopy analysis, we suspended 5 × 10^5^ NKCs in 1 mL 1 µg/mL carboxyfluorescein diacetate succinimidyl ester (CFSE; Dojindo Laboratories, Kumamoto, Japan) and incubated them at 37 °C for 30 min. We co-cultured the spheroids derived from 300 T98G and U251MG cells for 24 h with 3 × 10^3^ CFSE-labeled NKCs and observed them under a BZ-X700 all-in-one fluorescence microscope (Keyence, Osaka, Japan). We detected the CFSE-labeled NKCs with the green fluorescent protein filter (OP-87765, Keyence). The cells within all spheroids were visualized by recording merged Z-stack images using the BZ-X700 quick full-focus function.

For the flow cytometry-based apoptosis assay, we co-cultured 5 × 10^3^ cell-derived spheroids for 24 h with 5 × 10^4^ CFSE-labeled NKCs. Subsequently, we centrifuged and detached the cells with StemPro Accutase Cell Dissociation Reagent (Thermo Fisher Scientific) at 37 °C for 60 min. Then, we stained the cells with an allophycocyanin (APC)-conjugated Annexin V Apoptosis Detection Kit as per the manufacturer’s instructions (BioLegend, San Diego, CA, USA). We detected apoptotic GBM cells with a BD FACSCalibur flow cytometer (BD Biosciences) and analyzed them using FlowJo v10 (BD Biosciences). To ensure that the analysis was accurate, we gated out the CFSE-positive fraction to assess GBM cell apoptosis.

### In vivo orthotopic xenograft assays

We purchased 24 female non-obese diabetes/severe combined immunodeficiency/gc null (NOG) mice (6 weeks old) from the Central Institute for Experimental Animals (Kanagawa, Japan). The Institute of Animal Care and Use Committee of Nara Medical University approved all animal experiments. We anesthetized the mice with inhalation of isoflurane mixed with air (induction, 2.5%; maintenance, 1.5%) and fixed them on a stereotaxic instrument for mice (SR-6 M-HT, Narishige, Tokyo, Japan). We infused the mice stereotactically with 2 µL native Hank’s buffered salt solution (HBSS) that contained 10^5^ U87MG cells into the right thalamus (2 mm lateral and 2 mm posterior from the bregma and 3 mm dorsoventral from the outer cranium border) with a Hamilton syringe (33 S-gauge needle) mounted on an infusion syringe pump (Harvard Apparatus, Holliston, MA, USA). The mice were randomly assigned to three intracranial infusion groups (*n* = 8 mice per group): negative background (NB, HBSS only), NK mock, and NK dCIS (10^6^ cells). The mice were directly infused intracranially with the cells and reagents prepared using the aforementioned settings and using the infusion syringe pump via the same burr hole used for implanting the U87MG cells. The infusion speed for both the U87MG cells and NKCs was 1 µL/min.

### Histochemical analysis

We fixed the intracranial tumors in 10% neutral-buffered formalin, then embedded them in paraffin. We placed 5-µm thick sections on glass slides and stained them with hematoxylin–eosin (HE). We captured photographs at × 40 and × 400 magnification using a BX-710 microscope unit (Keyence). We performed the histochemical analyses using a BZ-X analyzer (Keyence).

### Statistical analysis

We performed statistical analysis with GraphPad Prism 8 (GraphPad Software, San Diego, CA, USA) and report the values as the mean ± SD or SEM. We determined the statistical significance of differences with the unpaired t-test, Mann–Whitney test, or one- or two-way analysis of variance (ANOVA), followed by Tukey’s or Sidak’s test. Statistical significance was accepted at *P* < 0.05. We estimated survival in each group mated using the Kaplan–Meier method, which encompassed the medians and OS rates. We determined the statistical significance of differences with log rank testing.

## Results

### Immunological feature in human GBM

**Fig. 1:**
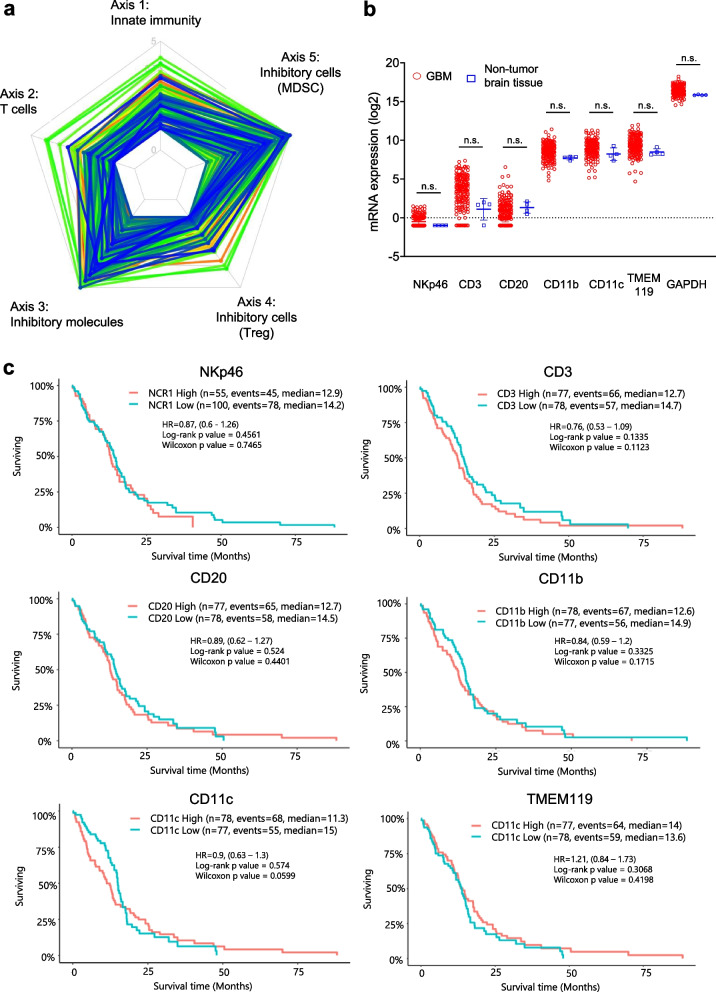
Immune cell-related gene expression in human GBM. **a** RNA-seq immunogram radar plots for human GBM. Five tumor immunity-related parameters were scored and plotted on the radar plot to depict the molecular immune profiles of the TME in 156 patients with GBM. Radar plot line colors denote each of the 156 samples. The radar plot consists of innate immunity (axis 1), T cell (axis 2), inhibitory molecules (axis 3), inhibitory cells (Treg, axis 4), and inhibitory cells (MDSC, axis 5). **b** NKp46, CD3, CD20, CD11b, CD11c, TMEM119, and GAPDH expression in GBM (*n* = 156 samples) and nontumor tissue (*n* = 4 samples). The data were from TCGA-based GlioVis analysis. Data are the mean ± SD. Statistical differences were determined by the Kruskal–Wallis test. n.s.: not significant. **c** Kaplan–Meier curves based on NKp46, CD3, CD20, CD11b, CD11c, and TMEM119 high and low expression. The data were from TCGA-based GlioVis analysis

The immunogram of 156 patients with GBM was obtained from TCGA-based Cancer Immunogram Web [31]. The immunogram ladder plot shapes depicted different but distinct trends for each patient. Most GBM cases had low T cell and innate immunity scores. Furthermore, the inhibitory molecule and inhibitory cell (MDSC; myeloid-derived suppressor cells) score was high but that for inhibitory cells (Treg; regulatory T cells) was low (Fig. 1a). MDSC and Treg are well-known as immunosuppressive cells with distinct different origins and systems that inhibit immunoactivity in GBM. Therefore, these cells should be examined separately. To determine the immune cell populations in GBM, NKp46 (NKC marker), CD3 (T cell marker), CD20 (B cell marker), CD11b (macrophage marker), CD11c (dendritic cell marker), TMEM119 (transmembrane protein 119; microglia marker), and GAPDH (internal positive control marker) expression was analyzed with GlioVis RNA-seq data. CD11b, CD11c, and TMEM119 were highly expressed, while NKp46 and CD20 expression was low and CD3 expression was relatively low and variable (Fig. 1b). These results indicated that GBM tumors lacked effector cells (T cells and NKCs) and had an immunosuppressive microenvironment based on the MDSC and microglia, which is known as an “immunologically cold” tumor feature. Moreover, the NKp46, CD3, CD20, CD11b, CD11c, and TMEM119 expression levels did not predict OS in the GlioVis database (Fig. 1c). These data suggested that a strong trigger is needed to induce an immune response in GBM.

### Induction of human primary NK dCIS by CRISPR/Cas9 RNP

**Fig. 2:**
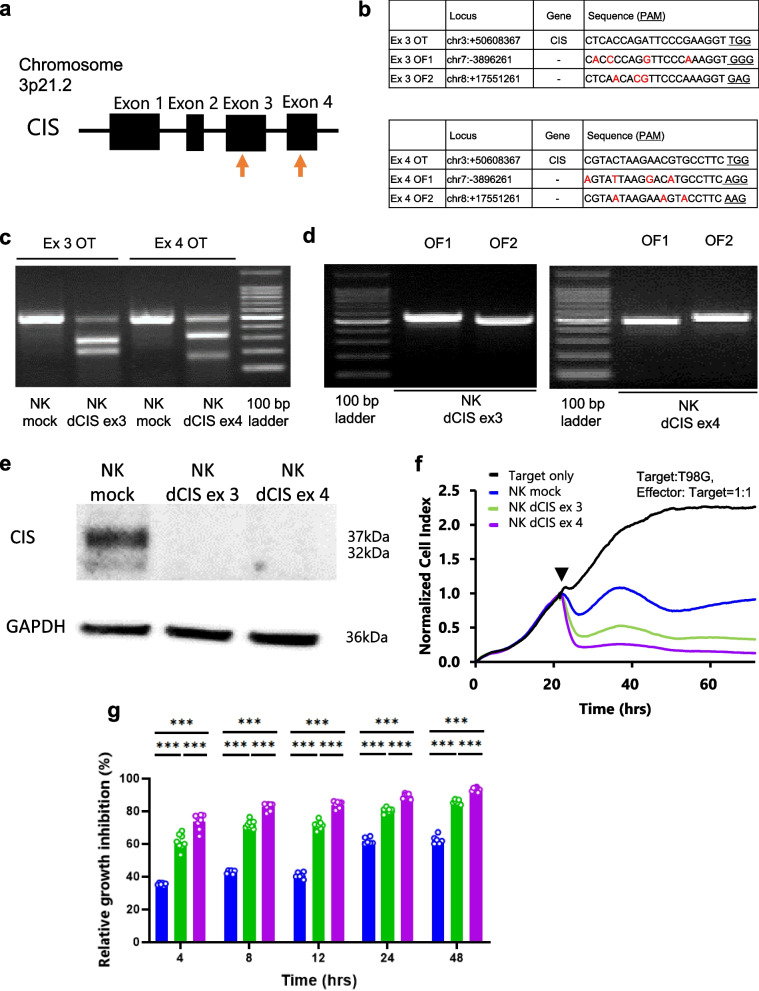
Design of sgRNAs targeting the *CIS* gene. **a** Schematic representation of the *CIS* gene. Orange arrows indicate sgRNA targeting sites. **b** OT (on target: top table) and OF (off target: bottom table) sequences of the *CIS* exon 3- and exon 4-targeting sgRNAs, respectively. Underlined letters in the target sequences indicate the protospacer adjacent motif (PAM) sequence. Red letters indicate sequences that differ from the target sgRNAs. **c** OT effects in the genome-edited NKCs. NKC genomic DNA was isolated and PCR was performed using primers flanking the OT region (Ex3 OT and Ex4 OT). The PCR product was reacted with T7E1. NK mock, NK dCIS ex3, and NK dCIS ex 4 indicate sgRNA/Cas9, *CIS* exon 3-targeting sgRNA/Cas9, and *CIS* exon 4-targeting sgRNA/Cas9-electroplated NKC DNA, respectively. **d** OF detection by T7E1 assays. PCR was performed using primers flanking the OF region. OF sites were predicted by an off-targeting potential checking system based on a homology-based algorithm. The top two expected sites were analyzed. Left and right photos depict the *CIS* exon 3 and exon 4-targeting sgRNAs, respectively, in the genomic DNA of the NK mock and NK dCIS. **e** CIS protein expression in genome-edited NKCs expanded from human peripheral blood. Top and bottom blots depict CIS protein and GAPDH protein, respectively. NK dCIS ex 3 and NK dCIS ex 4 were electroporated to exon 3 and exon 4, respectively, of the *CIS*-targeting sgRNA, tracrRNA, and Cas9 complex (RNP) and cultured for 7 days (total, 14-day culture). **f** Representative graphs of RTCA-based growth inhibition assay. The X- and Y-axes represent the co-culture duration and normalized cell index, respectively. The black arrowhead indicates the start of the culture. Black, blue, green, and purple lines indicate target only, NK mock, NK dCIS ex 3, and NK dCIS ex 4, respectively. The NKC-to-T98G cell co-culture ratio was 1:1 (2 × 10^4^:2 × 10.^4^ per well). **g** Graphs depicting relative growth inhibition of genome-edited NKCs. The X- and Y-axes represent co-culture duration and relative growth inhibition effects, respectively. Blue, green, and purple lines indicate NK mock, NK dCIS ex 3, and NK dCIS exon 4, respectively. Data are the mean ± SD. Statistical differences were determined by two-way ANOVA following Tukey’s test. At least two independent experiments were performed. *n* = 6, ***P < 0.001

We designed two sgRNAs targeting exon 3 and 4 of the *CIS* gene (Fig. 2a). CD3-depleted PBMCs obtained from volunteer were cultured for 7 days. Then, RNPs were created by incubating the designed sgRNA and tracrRNA with recombinant Cas9 prior to electroporation and guided into the in vitro expanded primary human NKCs. The NKCs were cultured for 7 days to allow sufficient time for protein turnover. The predicted OT and OF effects were detected with a T7E1-based mutation detection assay on the RNP-electroporated NKCs on day 7 after electroporation. Figure 2b lists the OT and OF site sequences. The assays demonstrated that both sgRNAs cleaved the target gene region of *CIS* (Fig. 2c), which clearly demonstrated the OT effects. The OF sites were predicted with a homology-based off-targeting potential detection system in the CRISPR/Cas9 system targeting exon 3 and 4 (Fig. 2b). The assays demonstrated that both sgRNAs cleaved the target gene region, which did not show the OF effects as long as we tested (Fig. 2d).

Western blotting revealed that both sgRNAs depleted *CIS* expression almost completely (Fig. 2e). Therefore, the CRISPR/Cas9 genome editing method we established was effective for disrupting CIS on the primary human NKCs. The real-time cell growth inhibition assays revealed that the NK dCIS electroplated by the target sgRNAs for *CIS* exon 3 and 4 significantly inhibited the growth of T98G cells, an NK activity-sensitive cell line, as compared to the NK mock. Furthermore, the sgRNA targeting *CIS* exon 4 significantly inhibited T98G cell growth as compared to that targeting exon 3 (Fig. 2f, g). These results indicated the successful induction of NK dCIS with two different *CIS* gene sgRNA target sites, and *CIS* deletion in the NKCs enhanced the growth inhibition of the NK activity-sensitive T98G cells. As the *CIS* exon 4-targeted sgRNA induced better inhibitory effects on GBM cells, it was selected for the subsequent experiments.

### Characterization of NK dCIS

**Fig. 3:**
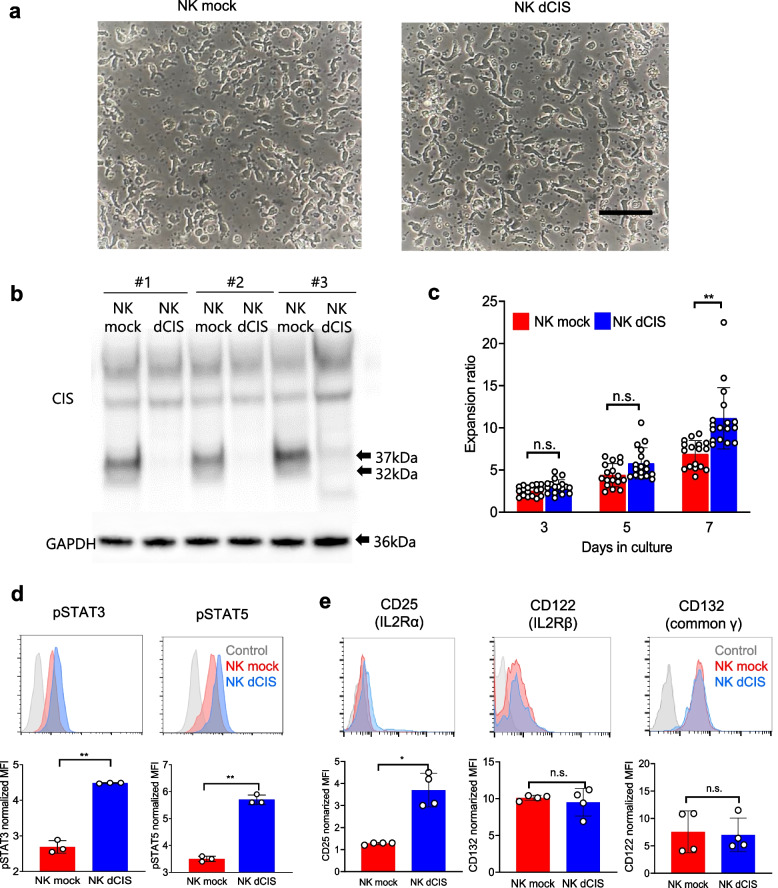
CRISPR/Cas9 Induction of NK dCIS. **a** NKC morphology under an inverted microscope. Left and right photographs depict NK mock and NK dCIS edited by *CIS* exon 4-targeting sgRNA, respectively. Black bar = 100 µm. **b** *CIS* expression in genome-edited NKCs. The RNP-electroporated NKCs were collected from three independent experiments and western blotting was performed. Top: The 32- and 37-kDa bands indicate the CIS protein. Bottom: GAPDH. **c** The NKC expansion ratio at 3, 5, and 7 days after electroporation. Data are the mean ± SD (*n* = 17). The significance of differences was determined by one-way ANOVA followed by Tukey’s test. n.s.: not significant. **P < 0.01. Data are from at least two independent experiments. **d** (Top) Representative histograms showing enhanced phosphorylation of STAT5 (pSTAT5) and STAT3 (pSTAT3) in NK mock vs. NK dCIS. Blue, red, and gray represent NK dCIS, NK mock, and negative background (NB) cells, respectively. (Bottom) Graphs depicting the normalized mean fluorescence intensity (MFI). Red and blue bars represent NK mock and NK dCIS, respectively (bottom). Data are the mean ± SD, *n* = 4. The significance of differences was determined by the unpaired t-test. **P < 0.01. **e** (Top) Representative histograms depicting IL-2 receptor expression on genome-edited NKCs. Blue, red, and gray represent NK dCIS, NK mock, and NB cells, respectively. (Bottom) Normalized MFI. Blue and red bars indicate NK mock and NK dCIS, respectively. Data are the mean ± SD, *n* = 4. The significance of differences was determined by the unpaired t-test. n.s.: not significant, *P < 0.05, **P < 0.01. All data were obtained from at least two independent experiments

The NK dCIS were characterized in detail. The RNP electroporation and transduction demonstrated no morphological changes on day 7 after electroporation (Fig. 3a). CIS expression was almost completely inhibited in the NK dCIS reproducibly (Fig. 3b) and the expansion ratio was increased in the NK dCIS as compared to the NK mock (Fig. 3c). Additionally, our NKC expansion method consistently induced 2–4 × 10^7^ NKCs from 16 mL human peripheral blood over 7-day culture. CD3-depleted NKCs were amplified by approximately 10 times following the additional 7-day culture after electroporation. Ultimately, 16 mL peripheral blood yielded 2–4 × 10^8^ genetically modified NKCs over 2 weeks. Flow cytometric analysis detected significantly increased STAT3 and STAT5 phosphorylation in the NK dCIS as compared to the NK mock (Fig. 3d). Expression analysis demonstrated that the NK dCIS had significantly increased IL-2 receptor α expression but that IL-2 receptor β and common ɤ receptor expression was not altered as compared to the NK mock (Fig. 3e).

Next, NK activating and inhibitory receptor expression on the NK dCIS was confirmed (Supplementary Fig. 1a). All NK receptors tested were highly or lowly expressed on the expanded NKCs. Specifically, PD-1 expression was extremely low. In the NK dCIS, NK activating receptors CD11a, DNAM-1, NKG2D NKp30, NKp46, and CD16 were not altered, but NKp44 was slightly upregulated, while the NK inhibitory receptors PD1, TIM-3, TIGIT, CD96, NKG2A, and KIR were not altered but LAG-3 was slightly upregulated (Supplementary Fig. 1b).

### Comprehensive differential gene expression microarray analysis in NK dCIS

**Fig. 4:**
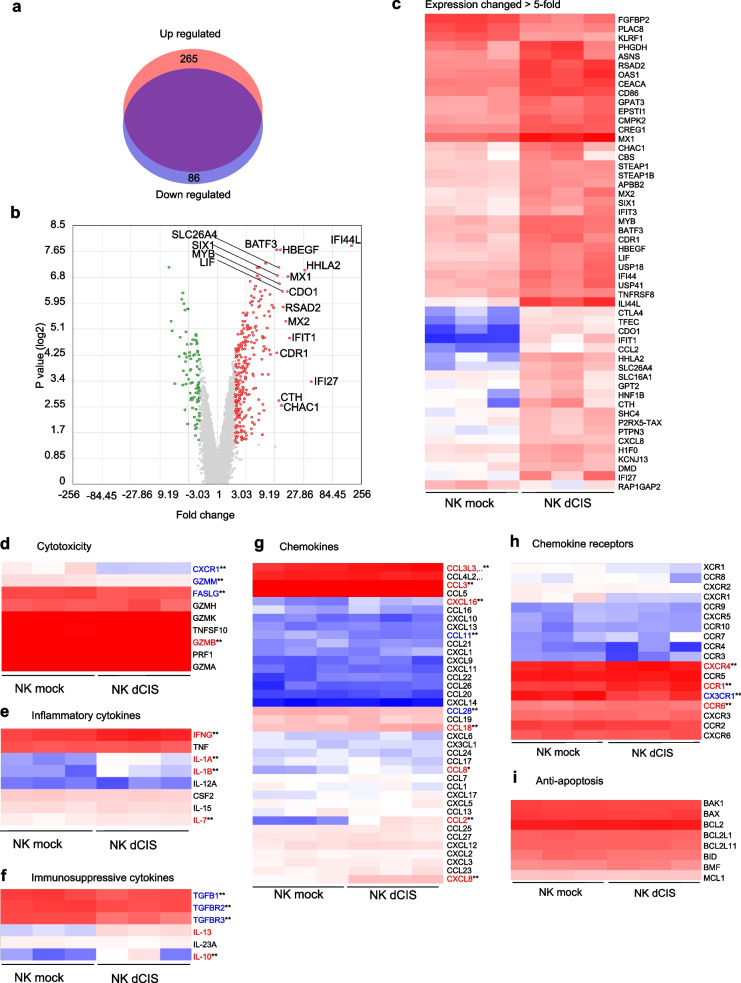
Comprehensive gene expression profiles of NK dCIS. **a** Upregulated (red) and downregulated (blue) genes in the NK dCIS. The purple region indicates unchanged genes. Fold change > 2 and > -2 and *P* < 0.05 indicated significant change. **b** Volcano plot analysis. Red and green dots indicate fold change > 2 and > -2, respectively, at *P* < 0.05. Genes with > tenfold change in expression are labeled. **c** Heatmap depicting genes with fold change > 5 or < -5 in NK dCIS as compared to NK mock with scaled intensity. Red and blue tones denote increased and decreased gene expression, respectively, as compared to the NK mock. **d–i** Heat maps representing genes clustered in cytotoxicity (**d**), inflammatory cytokine (**e**), immune suppression (**f**), chemokines (**g**), chemokine receptors (**h**), and anti-apoptosis (**i**) with scaled intensities under GOBP (Gene Ontology Biological Process). Red and blue tones denote increased and decreased gene expression, respectively, as compared to the NK mock. The significance of differences was determined by the t-test. *n* = 3, **P < 0.01. All data were from at least two independent experiments

We performed microarray analysis as an unbiased approach to identify the altered gene expression that enhanced the functions of NK dCIS and transcriptionally compared NK mock and NK dCIS from 7-day culture after electroporation. In total, more than 20,800 genes were evaluated, where 265 and 86 genes in the NK dCIS were upregulated and downregulated, respectively (Fig. 4a). The volcano plot demonstrated that fold change > 10 in the expression of 17 genes: *IFI44L* (interferon [IFN]-induced protein 44-like), *IFI27* (IFN, alpha-inducible protein 27), *HHLA2* (HERV-H LTR-associating 2), *HBEGF* (heparin binding-epidermal growth factor-like growth factor), *CDR1* (cerebellar degeneration-related 1), *BATF3* (basic leucine zipper ATF-like transcription factor 3), *IFIT1* (IFN-induced protein with tetratricopeptide repeats 1), *MX1* (MX dynamin-like GTPase 1), *MX2*, *CDO1* (cysteine dioxygenase type 1), *RSAD2* (radical S-adenosyl methionine domain containing 2), *LIF* (leukemia inhibitory factor), *CHAC1* (ChaC glutathione-specific gamma-glutamylcyclotransferase 1), *MYB* (v-myb avian myeloblastosis viral oncogene homolog), *SLC26A4* (solute carrier family 26 (anion exchanger), member 4), *CTH* (cystathionine gamma-lyase), *SIX1* (SIX homeobox 1) (Fig. 4b). The heatmap depicts the genes with fold change > 5 or < -5 in the NK dCIS as compared to the NK mock with scaled intensity (Fig. 4c). *IFI27*, *IFIT1*, *MX1*, *MX2*, and *RSAD2* were significantly upregulated in the NK dCIS, which suggested that deleting *CIS* in the expanded NKCs enhanced the expression of IFN-related genes.

Among the cytotoxicity-related genes, *GZMB* (granzyme B) was upregulated and *FASLG* (Fas ligand), *GZMM* (granzyme M), and *CXCR1* were downregulated in the NK dCIS. Among the inflammatory cytokines, *IFNG* (IFNɤ) *IL1A*, *IL1B*, and *IL7* were upregulated in the NK dCIS. Among the immunosuppressive cytokines and related molecules, *TGFB1* (tumor growth factor β1), *TGFBR2* (TGFB receptor 2), and *TGFBR3* (TGFB receptor 3) were downregulated while *IL13* and *IL10* were upregulated in the NK dCIS. Among the chemokine genes, *CCL3L3*, *CCL3*, *CXCL16*, *CCL18*, *CCL8*, *CCL2*, and *CXCL8* were upregulated and *CCL11* and *CCL28* were downregulated in the NK dCIS. Among the chemokine receptors, *CXCR4*, *CCR1*, and *CCR6* were upregulated and *CX3CR1* was downregulated in the NK dCIS. The expression of the anti-apoptosis genes was not altered in the NK dCIS (Fig. 4d–i).

**Fig. 5:**
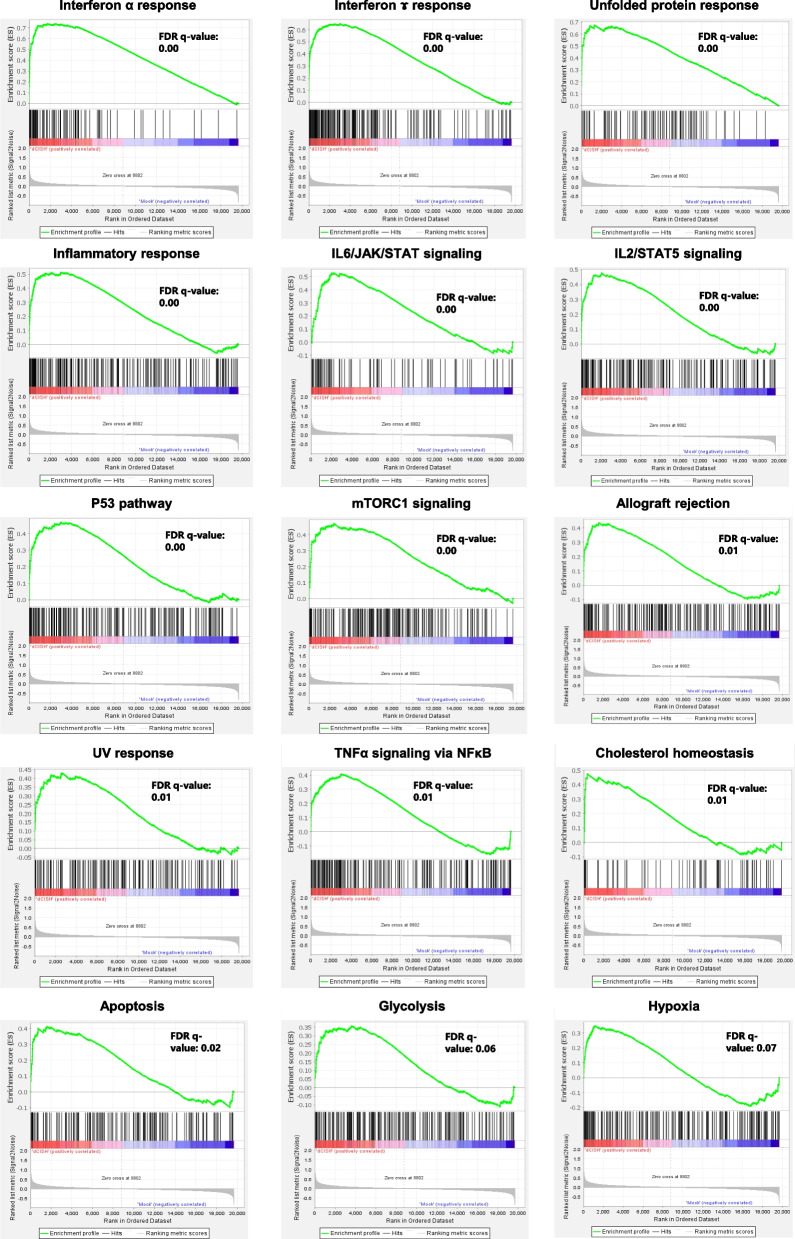
GSEA of NK dCIS. Representative GSEA plot depicting enrichment in IFNα response, IFNɤ response, unfolded protein response, inflammatory response, IL-6–JAK–STAT signaling, IL-2–STAT5 signaling, p53 pathway, mTORC1 signaling, allograft rejection, UV response, TNFα signaling via NFκB, cholesterol homeostasis, apoptosis, glycolysis, and hypoxia. (Top) X-axis indicates NK dCIS-correlated genes (left) and NK mock-correlated genes (right), represented by bar code data. Y-axis indicates the enrichment score. Bottom: X- and Y-axes indicate the rank in ordered dataset and ranked list metrics, respectively. A false discovery rate q value < 0.05 indicated a significant difference

The GSEA plots demonstrated the enrichment of effector functions (inflammatory response, allograft rejection, IFNα response, IFNɤ response, IL-6–JAK–STAT signaling, IL-2–STAT5 signaling, mammalian target of rapamycin [mTOR] complex 1 [mTORC1] signaling, tumor necrosis factor [TNFα] signaling via NFκB), gene repair-related responses (UV response and the p53 pathway), apoptosis-related genes, and metabolic pathways (cholesterol homeostasis-related genes). The glycolysis-related genes tended to be enriched but not significantly changed. Lastly, the genes for the unfolded protein response, a cellular stress response mechanism activated when cells encounter an accumulation of unfolded or misfolded proteins in the endoplasmic reticulum, were enriched (Fig. 5).

Above raw data of differential gene expression were shown in Supplemental data 1.

### Functional characterization of NK dCIS on human allogeneic GBM cells in vitro

**Fig. 6:**
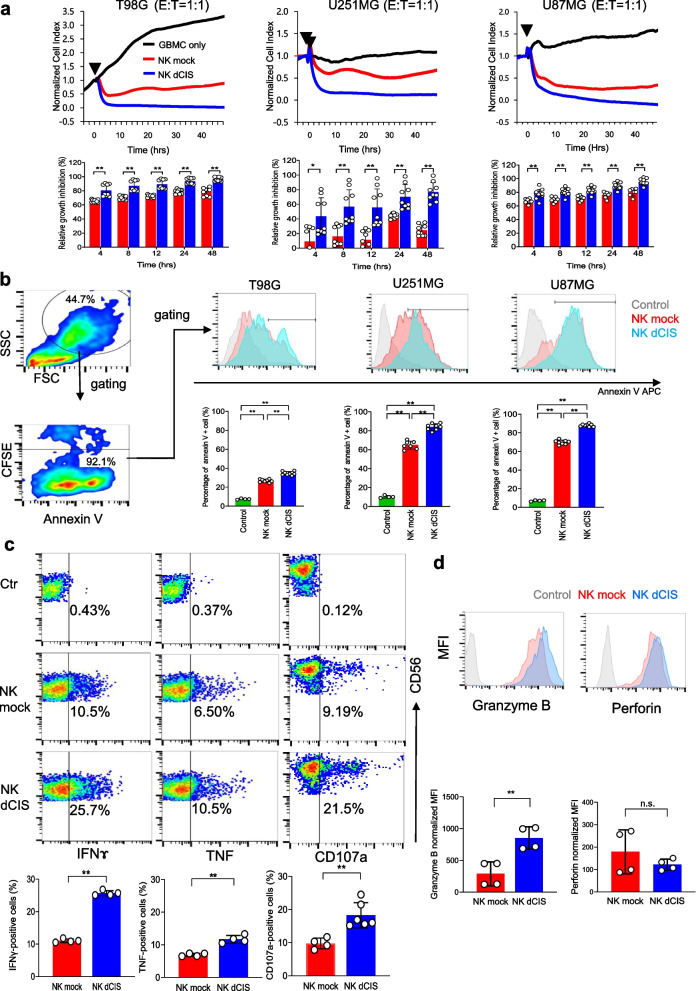
*CIS* deletion enhances effector function of the expanded NKCs on allogeneic GBM cells. **a** (Top): Representative graphs of the RTCA-based growth inhibition assay. X- and Y-axes denote the co-culture duration and normalized cell index, respectively. The normalized cell index indicates which NKC addition time was selected and set by the software as 1.0, based on which all values are depicted as a percentage. Black arrowhead indicates the start of the culture. The target cells were T98G, U251MG, and U87MG cells. Black, red, and blue curves indicate target only, mock NKCs, NK dCIS, respectively. The effector (NKCs)-to-target cell (T98G, U251MG, U87MG) co-culture ratio was 1:1 (2 × 10^4^:2 × 10^4^ per well). (Bottom) Graphs depicting relative growth inhibition of NK dCIS. X- and Y-axes denote co-culture duration and relative growth inhibition effects, respectively. Red and blue bars indicate NK mock and NK dCIS, respectively. Data are the mean ± SD. Statistical differences were determined by two-way ANOVA followed by Tukey’s test. *n* = 5–10, **P < 0.01. **b** Flow cytometry-based apoptosis assay. The target cells were T98G, U251MG, and U87MG cells. The NKC-to-target cell co-culture ratio was 1:1. The co-culture spanned 24 h. (Left) Red and blue color maps indicate high and low density, respectively. (Top right) Histograms depict annexin V-positive cells gated by CD45-negative fractions. Gray, red, and blue histograms represent the control, NK mock, and NK dCIS, respectively. (Bottom right) Graphs illustrate the percentage of annexin V-positive cells. Statistical differences were determined by one-way ANOVA followed by Tukey’s test. *n* = 4–7, **P < 0.01. **c** Cytokine production assays and evaluation of CD107a expression in NKCs. The NKCs were co-cultured with T98G cells for 5 h. Representative red and blue color maps gated by the CD56-positive fraction denote high and low density, respectively. Y-axes denote CD56 positivity. X-axes denote IFNɤ (left), TNF (center), and CD107a (right) expression. (Top) Graphs denote IFNɤ (left), TNF (center), and CD107a (right) positivity. Red and blue bars indicate NK mock and NK dCIS, respectively. Data are the mean ± SD. Statistical differences were determined by the t-test or Mann–Whitney U test. *n* = 4–6, **P < 0.01. **d** Expression of cytotoxic granules in NKCs. (Top) Representative histograms gated by the CD56-positive fraction. Gray, red, and blue bars indicate control, NK mock, and NK dCIS, respectively. (Bottom) Graphs depict granzyme B and perforin expression in NKCs. Statistical differences were determined by the t-test or Mann–Whitney U test. *n* = 4, n.s.: not significant, **P < 0.01. At least two independent experiments were performed

The functional aspects of the NK dCIS were characterized on allogeneic GBM cells in vitro. RTCA-based growth inhibition assays revealed that the NK dCIS inhibited T98G, U251MG, and U87MG cell growth time-dependently as compared to the NK mock (Fig. 6a). Furthermore, NK dCIS derived from two different donors were successfully induced and enhanced NKC-mediated growth inhibition, suggesting no difference in these effects between donors (Supplementary Fig. 2). The annexin V-based flow cytometry apoptosis assays demonstrated that the NK dCIS enhanced T98G, U251MG, and U87MG cell apoptosis as compared to the NK mock (Fig. 6b). These data indicated that apoptosis enhanced the growth inhibitory effects of the NK dCIS.

Cytokine production assays demonstrated that *CIS* deletion in the NKCs enhanced IFNɤ and TNF production on allogeneic GBM cells. The NK dCIS had upregulated expression of the cytotoxicity marker CD107a (LAMP1: lysosomal-associated membrane protein 1) (Fig. 6c). The cytotoxic granules of the NK d*CIS* contained increased amounts of granzyme B, but perforin was unchanged (Fig. 6d).

### Apoptosis induction effects of NK dCIS in GBM cell-derived spheroids

**Fig. 7:**
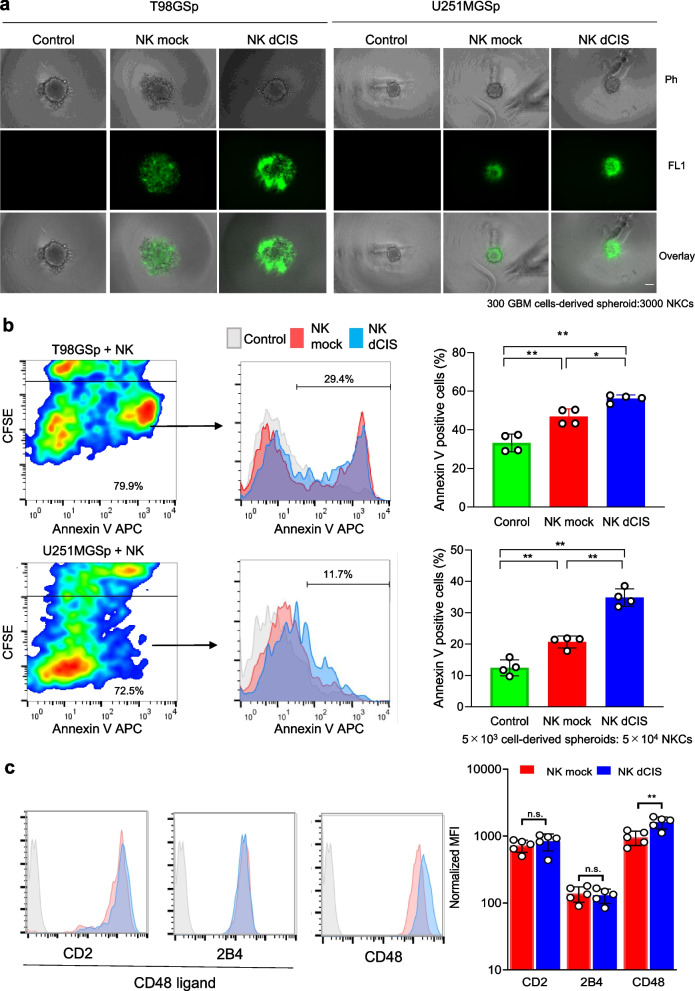
*CIS* deletion enhances apoptosis in GBM spheroids. **a** Fluorescent microscopic evaluation of T98G- and U251MG-derived spheroids co-cultured with NKCs. Scale bars = 100 μm. GBM cells (300 cells/well) were seeded onto nonadherent V-bottom 96-well plates for 1 day, co-cultured with 3 × 10^3^ CFSE-labeled NKCs, and observed under a BZ-X700 fluorescence microscope. The cells within all spheroids were visualized by recording merged Z-stack images using the BZ-X700 quick full-focus function. Phase contrast (top, Ph), FL1-based fluorescent (middle, FL1), and overlay (bottom, overlay) images are shown. **b** Flow cytometric analysis of the apoptosis of T98G- and U251MG-derived spheroids co-cultured with NKCs. The spheroids (5 × 10^3^) were co-cultured for 24 h with 5 × 10^4^ CFSE-labeled NKCs. Subsequently, the cells were centrifuged and detached with Accutase, then stained with APC-conjugated annexin V. Apoptotic GBM cells were detected with a flow cytometer. To ensure that the analysis was accurate, the CFSE-positive fraction was gated out to assess GBM cell apoptosis. (Left) Flow cytometry-based apoptosis assay of T98G (top) and U251MG cells (bottom). Red and blue color maps indicate high and low density, respectively. (Middle) Histograms negatively fractionated by FL-1-positive cells (CFSE) depict annexin V-positive cells. X-axis denotes APC-conjugated annexin V. (Right) Graphs indicate the percentage of annexin V-positive GBM cells. Data are the mean ± SD of four experiments. Statistical differences were determined by two-way ANOVA followed by Tukey’s test. *P < 0.05, **P < 0.01. **c** Adhesion molecule expression in NKCs. (Left) Histograms depict CD2, 2B4, and CD48. CD2 and 2B4 are CD48 ligands. Gray, red, and blue histograms represent the control, NK mock, and NK dCIS, respectively. (Right) Graph depicts normalized MFI. Red and blue bars indicate NK mock and NK dCIS, respectively. Data are the mean ± SD of five experiments. Statistical differences were determined by the t-test. n.s.: not significant, *P < 0.05, **P < 0.01. At least two independent experiments were performed

We evaluated the apoptosis induction effect of the NKCs in GBM spheroids by flow cytometry to confirm the enhanced cytotoxicity of the NK dCIS. Fluorescent microscopic analysis of NKC dynamics against GBM spheroids revealed that the NKCs accumulated around the spheroids, where the NK dCIS formed large clusters as compared to the NK mock (Fig. 7a). Annexin V-positive apoptotic cells in the GBM spheroids were analyzed by gating out the CD45-negative fractions. The NK mock and NK dCIS respectively induced 43.2–50.4% and 53.4–57.8% annexin V-positive cells among the T98G cells and 17.8–21.8% and 31.9–38.5% annexin V-positive cells among the U251MG cells. The NK dCIS induced significantly higher GBM spheroid apoptosis compared to the NK mock (Fig. 7b). The expression of CD2, 2B4 (NKC cell adhesion factors), and its ligand CD48 was evaluated to confirm the formation of large cell clusters in the NKCs. CD48 expression was significantly upregulated in the NK dCIS, suggesting that CD48 might be involved in the formation of large cell clusters (Fig. 7c). These results supported the idea that disrupting *CIS* was crucial for enhancing NKC activity against GBM spheroids.

### *CIS* deletion enhanced NKC anti-tumor activity in the allogeneic brain tumor model

**Fig. 8:**
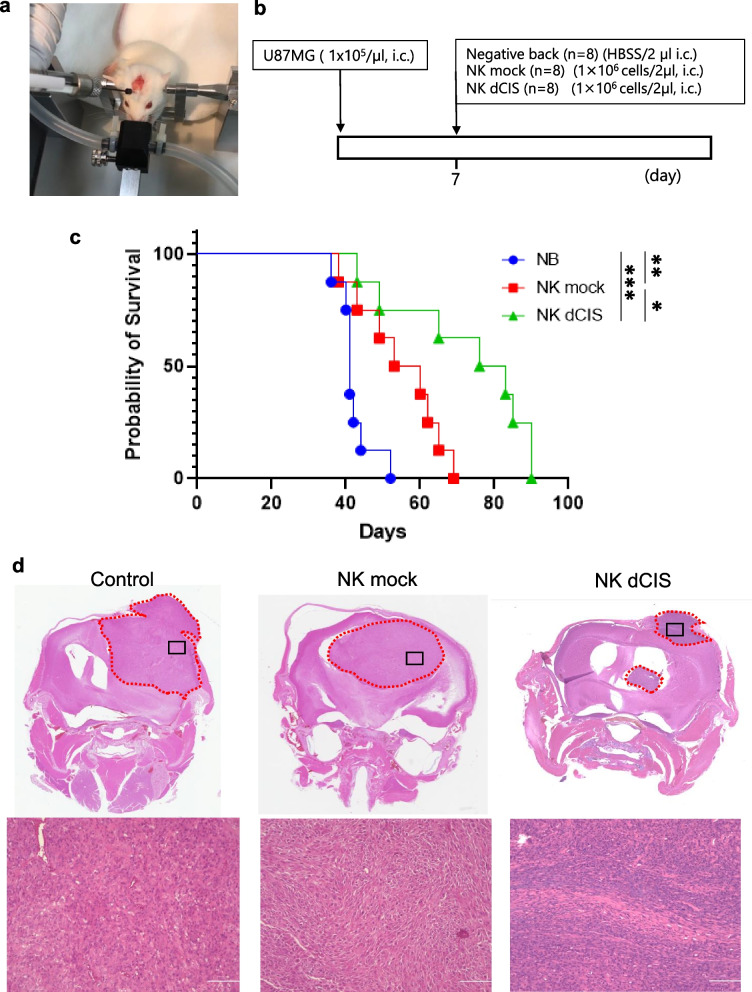
NK dCIS prolong overall survival in an allogeneic xenograft brain tumor model. **a** Photo depicts injection of U87MG cells into NOG mouse brain. **b** Schematic of the experimental design. **c** Graph depicting the Kaplan–Meier curve. Blue, red, and green lines represent the NB (*n* = 8; HBSS/2 µL), NK mock (*n* = 8; 1 × 10^6^ NK mock/2 µL), and NK dCIS (*n* = 8; 1 × 10^6^ NK dCIS/2 µL), respectively. Survival in each group was estimated using the Kaplan–Meier curve method. The statistical significance of differences was determined using the log rank test. ***P < 0.001, **P < 0.01, ***P < 0.05. **d** Photo of HE staining. Top and bottom images depict × 40 and × 400 magnification, respectively. Representative histocytological features at the time of autopsy of NB (left), NK mock (center), and NK dCIS (right) tumors are shown. Black squares in × 40 photos indicate the area observed under × 400 magnification

The mechanistic insights gained from the above studies suggested that deleting *CIS* from NKCs regulates NKC cytotoxic activity and therefore could provide a useful target for GBM immunotherapy. To evaluate the hypothesis, we tested the anti-tumor effects of allogeneic NK dCIS in intracranial orthotopic xenografts derived from U87MG using NOG mice. U87MG cells were implanted into NOG mouse brains, followed by intracranial infusion through the same burr hole used for U87MG implantation (Fig. 8a, b). The control NK mock group was significantly associated with longer survival time compared to the NB group (mOS: 41.0 days vs. 56.5 days). The NK dCIS group exhibited prolonged OS compared to the NK mock group (mOS: 79.5 days) (Fig. 8c). The histochemical analysis revealed that the tumors from all groups exhibited human GBM histological features at the time of autopsy (Fig. 8d).

## Discussion

Conventionally, adoptive immunotherapy using lymphokine-activated killer cells, which are patient-derived high-dose IL-2-stimulated PBMCs, presented the possibility of a new treatment for patients with malignant glioma, including GBM. However, the anti-tumor effect against GBM was limited due to the low purity and expansion efficacy of NKCs [37]. To resolve this issue, patient PBMCs were co-cultured with the irradiated human renal cell carcinoma cell line HFWT and yielded high-purity NKCs with high expansion efficacy. Although the NKC-based immunotherapy was safe and partially effective in patients with recurrent malignant gliomas, the NKC expansion method was complicated and NKC purity varied between patients. Consequently, stable clinical trials were challenging and only a small-scale clinical trial was conducted [38]. We previously reported a feeder-free-based NKC expansion strategy that stably yielded therapeutic highly purified NKCs, which could be a promising therapy against GBM. However, the immunosuppressive GBM microenvironment, where few effector cells are present, might require an extremely strong trigger to induce an immune response for complete cure. Clinical trials demonstrated that allogeneic primary NKCs from human peripheral blood were safe without causing significant toxicity, such as CRS, neurotoxicity, or GVHD. Although allogeneic NKCs have potent anti-AML activity, their efficacy for treating solid tumors is limited [39–41]. Although our established NKC expansion method could be suitable for allogeneic NKC-based immunotherapy, further modification is necessary to obtain the therapeutic benefit in solid tumors, including GBM. A negative regulator of NKC function, CIS is a candidate for improving the anti-tumor effects of allogeneic NKCs in GBM.

A previously reported human primary NKC expansion system used irradiated autologous PBMCs [42]. In the past, human primary NKCs were expanded with various NKC expansion methods, which included co-culturing with the HFWT Wilms tumor cell line [38], gene-modified K562 leukemia cells [43], irradiated PBMCs [44], or using expanded T cells as feeder cells [14]. However, using feeder cells for NKC expansion is complex and susceptible to viral or bacterial infections, including mycoplasma. Although stimulation with OK432, a dead form of *Streptococcus pyogenes*, was reported, the system demonstrated inconsistent expansion efficiency and purity [45, 46]. Furthermore, confirming sterility in this system is challenging because the presence of viable or dead bacteria cannot be accurately determined during the culture process. Being able to safely and consistently expand numerous PBMC-derived NKCs without using feeder cells or dead bacteria could be the most advantageous approach for the clinical application of NK dCIS-based immunotherapy for cancer. The NKC expansion system we established is capable of yielding large numbers of highly purified NKCs without the use of feeder cells [30]. Moreover, this simple and sophisticated technology enabled us to produce a technology to induce a high amount of NK dCIS, which require more complex manipulations. In the present study, we used CRISPR/Cas9 to successfully induce NK dCIS in combination with the NKC expansion system. We have genome-edited *TIM3* in human primary NKCs using this gene-editing technology [33], and the expansion efficacy and gene disruption efficiency were comparable in the NK dCIS. In this study, 10^8^ order NK dCIS were obtained from 16 mL human peripheral blood. We confirmed that the human primary NK dCIS demonstrated improved effector functions, including cytokine production and cytotoxicity. Although Rautela et al. reported on human NK dCIS and evaluated their cytotoxicity and in vivo persistence [28], the NKCs they expanded were purified from PBMCs and expanded over 3 days by IL-2 before electroporation was performed. Although they did not describe the NKC expansion efficacy, our NKC culture technique had a higher expansion rate than theirs. Therefore, we were able to obtain large amounts of NK dCIS and confirm detailed characteristics, including comprehensive gene expression analysis and the anti-tumor effect against GBM ex vivo and in vivo. To our knowledge, this is the first report of NK dCIS exerting anti-tumor effects on GBM ex vivo and in vivo via apoptosis induction.

In this study, microarray analysis was performed to analyze in detail the changes caused by knocking out the *CIS* gene. The mRNA expression of the receptors and cytotoxic granules whose protein expression was obtained by flow cytometric analysis are depicted in Supplemental Fig. 5. The IL-2RA, LAG-3, and GZMB protein and mRNA levels were upregulated, while NKp44 protein levels were slightly upregulated whereas its mRNA expression was stable. The mRNA and protein levels of the remaining 14 genes were mostly stable. The possible reason for the NKp44 mismatch result was the indirectly increased transcription efficiency due to the increased CIS expression. These results indicated good validation of the comprehensive gene expression analysis in the present study. Differential gene expression analysis revealed that *CIS* deletion regulated small-scale gene expression and GSEA revealed that the genes associated with IFNα response, IFNɤ response, IL-6–JAK–STAT signaling, IL-2–STAT5 signaling, mTORC1 signaling, and TNFα signaling via NFκB were involved in *CIS* deletion in the NKCs. Cellular metabolism is now recognized as vital in regulating immune cell function and differentiation [47–50]. Immune cells undergo dynamic metabolic shifts to support their activity, and recent studies demonstrated that this is specifically true in NKC function [51, 52]. For example, IFNɤ production via NK receptor activation requires glucose-driven oxidative phosphorylation [53]. Another study reported that mTORC1, a key cellular metabolism regulator, was robustly stimulated in activated NKCs and required for IFNɤ production [54]. mTOR is activated by IL-15 and is essential for IL-15-mediated NKC proliferation during development and activation [48, 55]. Additionally, both aberrant glucose metabolism [56] and lipid accumulation [57] in NKCs lead to dysfunction. Here, we confirmed that deleting *CIS* in human primary NKCs improved their effector functions via STAT3/5 phosphorylation, which might involve mTORC1 and p53 signaling. Furthermore, *CIS* deletion in the NKCs involved cholesterol metabolism and tended to involve glycolysis. Our results supported previous findings of CIS function in human NKCs.

GSEA demonstrated that the unfolded protein response-related genes were enriched in the NK dCIS. Endoplasmic reticulum stress occurs with endoplasmic reticulum lumen accumulation of proteins with abnormal higher-order structures or that are not normally modified, i.e., unfolded proteins. Such proteins are caused by various physiological stresses, such as calcium depletion in the endoplasmic reticulum, cellular oxidative stress, the expression of mutant proteins, and low-glucose or hypoxic conditions [58, 59]. As endoplasmic reticulum stress can damage cells, cells have a system to avoid it [60–62] via three cellular responses: 1) inhibiting mRNA translation to prevent new proteins from being transported into the endoplasmic reticulum [63]. 2) Inducing the transcription of endoplasmic reticulum molecular chaperones to increase protein folding efficiency [64, 65]. 3) Activating endoplasmic reticulum-associated degradation to degrade the unfolded proteins [66, 67]. We hypothesized that this intracellular mechanism occurs in the NK dCIS. Therefore, we examined *CIS* mRNA expression in the genome-edited NKCs and determined that deleting *CIS* significantly but weakly inhibited *CIS* mRNA expression in the NKCs despite the complete inhibition of CIS protein expression (Fig. 3b, Supplementary Fig. 3). These accumulating findings suggested that abnormal proteins could degrade in the endoplasmic reticulum of NK dCIS.

Deleting *CIS* the in NKCs elicited anti-tumor effects in ex vivo GBM spheroids and an in vivo xenograft model. Comparison of the two models revealed that the apoptosis-inducing effect on the spheroids was weaker than the in vivo anti-tumor effect on the xenograft mouse model. It is possible that the spheroids were only a mass of GBM cells and the NKCs could not penetrate the interior of the cell cluster and did not attack the inner cells directly, which would weaken the effect of the NK dCIS. Furthermore, the anti-tumor effect on the spheroids was evaluated only 24 h after co-culture. Therefore, it is unsurprising that the survival prolongation effects in the xenograft model and the apoptosis induction effect in the spheroids did not completely match.

In clinical application, NK dCIS are intended for intratumor administration, with as much tumor removed as possible after surgery. In the animal experiments, the NKCs were administered 7 days post-transplantation. It was confirmed that the NK dCIS extended the OS in the mice by approximately twofold as compared to the group that received no NKCs. However, the NK dCIS did not lead to a radical cure. Nevertheless, unlike NOG mice, immune cells exist in humans in vivo. NK dCIS injected into the brain could attract other immune cells to the brain. For example, NK dCIS recruit conventional type 1 dendritic cells (cDC1) by secreting the chemokines CCL5 and XCL1 [68]. CD4 T cells are primed by cDC1 and activate systemic immunity [69]. This response may exert a greater anti-tumor effect on GBM than initially assumed and allow checkpoint antibodies that primarily target T cells to exert their effects.

Pierre-Louis Bernard and colleagues conducted an excellent study on the role of CIS in NKCs. They reported that inhibiting CIS in mouse NKCs enhanced their proliferation, functions, and activation of important pathways. When CIS was absent from the living organism, NKCs infiltrated tumors more effectively, improved their ability to kill cancer cells, and reduced exhaustion. Consequently, primary tumor growth and cancer spread to other parts of the body were significantly impeded in the animal models. Furthermore, they combined lentiviral pseudotype and the CRISPRi-dCas9 tool to target CIS in a human leukemic NKC line (NK-92) and primary NKCs, which resulted in improved cell functionality. These findings validated CIS as a promising target to enhance NKC immunotherapy [70]. However, their experiments were mainly conducted in mice, and only NK receptor-stimulated signaling has been described for primary human NKCs.

As gene transfer to human NKCs via lentivirus is extremely inefficient, it yielded what we considered a low amount of dCIS primary human NKCs. Furthermore, when considering future clinical applications, they used feeder cells (EBV-LCL) to culture primary human NKCs, which are difficult to handle and unsuitable for clinical applications. In the present study, we were able to prepare 2–4 × 10^8^ NK dCIS from 16 mL human blood in 2 weeks using the feeder-free NKC culture technique we established and RNP electroporation. This approach enabled more detailed in vitro, ex vivo, and in vivo analyses of dCIS human primary NKCs, and specifically revealed anti-tumor effects on GBM. These results indicated that clinical trials using not only NK dCIS but also other checkpoint molecule-knockout NKCs for GBM would be feasible using our technology.

In this study, we evaluated the anti-tumor effect of allogeneic NKCs in GBM. In a clinical trial of GBM, NK dCIS could be induced from healthy human blood and stocked in advance, which would facilitate stable clinical studies. Furthermore, the survival period can be prolonged by increasing the number of times the NKCs are administered. Overall, NK dCIS are a highly feasible and effective GBM treatment. A limitation of the present study was that in the in vivo model, pathological sections were prepared after death to confirm the presence of NKCs, but the presence of NKCs was not confirmed. NKCs probably do not survive long in brain tumors, and considering the time of tumor formation, it would be difficult to confirm the presence of NKCs with the present experimental scheme. Therefore, it is necessary to increase the number of doses and analyze the presence of NKCs in the tumor during tumor formation.

## Conclusion

In conclusion, we successfully induced NK dCIS using CRISPR/Cas9 with efficient expansion. *CIS* deletion enhanced the NKC-mediated anti-tumor effects in allogeneic GBM. Therefore, allogeneic NK dCIS could be a promising immunotherapeutic alternative for patients with GBM.

### Supplementary Information

**Additional file 1.** 

**Additional file 2.** 

**Additional file 3:** **Supplementary Fig. 1. **Effect of *CIS* deletion on NKC receptor expression on the expanded NKCs. a Representative histogram of NK mock and NK dCIS analyzed by flow cytometry for NK activating and inhibitory receptors. Seven representative activating and inhibitory receptors are shown. The histograms were gated by the CD56-positive fraction. Blue, red, and gray histograms represent NK dCIS, NK mock, and negative background (NB) cells, respectively. b Graph depicts normalized MFI. Blue and red bars indicate NK mock and NK dCIS, respectively. Data are the mean ± SD, *n* = 4. The significance of differences was determined by the t-test or Mann-Whitney U test. n.s.: not significant, *P< 0.05. Data are from at least two independent experiments. **Supplementary Fig. 2. **Induction of NK dCIS from two independent volunteers. (Left and right) NK dCIS data from Volunteer 1 and 2, respectively. (Top) Graph shows the NKC expansion ratio 3, 5, and 7 days after electroporation. Data are the mean ± SD (*n* = 5). The significance of differences was determined by one-way ANOVA followed by Tukey’s test. n.s.: not significant, **P <0.01, *P < 0.05. (Middle and bottom) Growth inhibition assays of NK mock and NK dCIS on T98G (middle) and U251MG cells (bottom). E:T ratios were 0.5:1 and 1:1 (0.5 × 10^6^:1 × 10^6^ and 1 × 10^6^:1 × 10^6^), respectively. Red, pink, green, light blue, and dark blue lines indicate GBM cells only (E:T = 0:1), NK mock (E:T = 0.5:1), NK dCIS (E:T = 0.5:1) NK mock (E:T = 1:1), and NK dCIS (E:T = 1:1), respectively. Data are the mean ± SD (*n *= 3–4). **Supplementary Fig. 3. ***CIS* mRNA expression in the CIS protein-deleted NKCs. Graph shows mRNA expression in the CIS protein-deleted NKCs extracted from microarray data. Blue and red graphs denote NK mock and NK dCIS, respectively. Data are the mean ± SD (*n* = 3). The significance of differences was determined by the t-test. *P< 0.05. **Supplementary Fig. 4. **The influence of CIS expression and OT/OF effects in NK dCIS 14 days after electroporation of *CIS* exon 4 targeting-RNP. a Western blot analysis of CIS protein expression (top) and GAPDH expression (bottom) in NK mock and NK dCIS 14 days after RNP electroporation. CIS protein position in electrophoresis is indicated at 32 and 37 kDa. b OT (top) and OF (bottom) effects in in NK mock and NK dCIS 14 days after RNA electroporation. Right lanes depict 100-bp marker DNA. **Supplementary Fig. 5. **Representative mRNA expression values in NK mock and NK dCIS obtained by Clariom™ S array microarray. The mRNA expression of the receptors and cytotoxic granules whose protein expression (in parentheses) was obtained by flow cytometric analysis are as follows: IL-2RA (CD25), IL2RB (CD122), IL2RG (CD123), GZMB (granzyme B), PFR1 (perforin), ITGAL (CD11a), CD226 (DNAM-1), KLRK1 (NKG2D), NCR1 (NKp46), NCR2 (NKp44), NCR3 (NKp30), FCGR3A (CD16), PDCD1 (PD1), LAG3 (LAG3), HAVCR2 (TIM3), TIGIT (TIGIT), CD96 (CD96), and KLRC1 (NKG2A). The mRNA signal intensity (log_2_) was corrected by calculating the characteristic control signal values. The significance of differences was determined by the t-test. *n* = 3, **P < 0.01. All data were from at least two independent experiments.

## Data Availability

The datasets generated during the current study are available from the corresponding author upon reasonable request.
