# Supplementary figures and images for: CIS deletion by CRISPR/Cas9 enhances human primary natural killer cell functions against allogeneic glioblastoma
Source: J Exp Clin Cancer Res. 2023 Aug 10;42:205. doi: 10.1186/s13046-023-02770-6 (PMC10413513; doi:10.1186/s13046-023-02770-6)

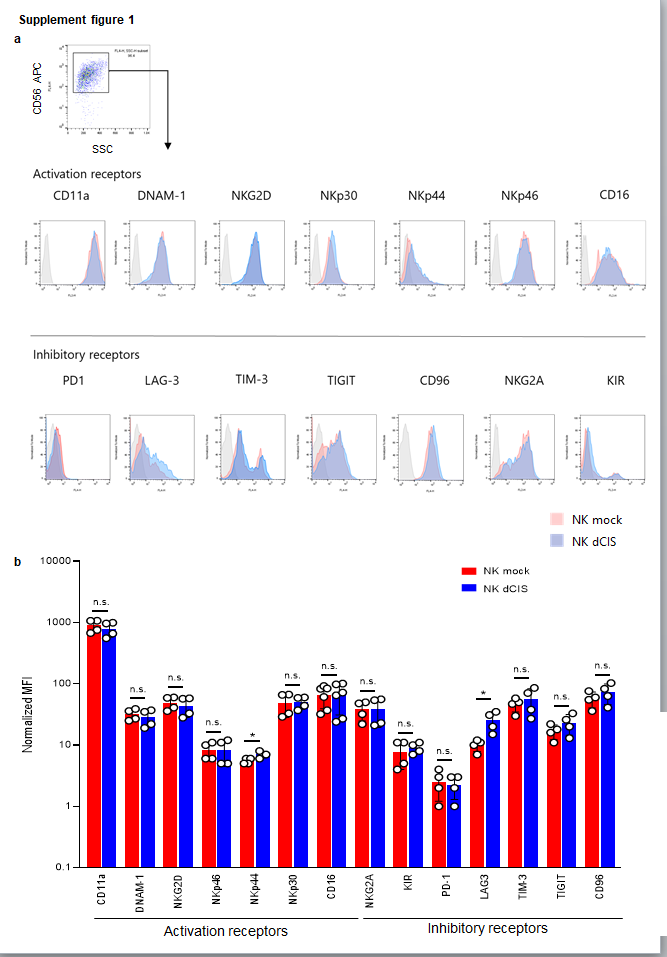

Supplement: Supplementary file 3 — Additional file 3: Supplementary Fig. 1. Effect of CIS deletion on NKC receptor expression on the expanded NKCs. a Representative histogram of NK mock and NK dCIS analyzed by flow cytometry for NK activating and inhibitory receptors. Seven representative activating and inhibitory receptors are shown. The histograms were gated by the CD56-positive fraction. Blue, red, and gray histograms represent NK dCIS, NK mock, and negative background (NB) cells, respectively. b Graph depicts normalized MFI. Blue and red bars indicate NK mock and NK dCIS, respectively. Data are the mean ± SD, n = 4. The significance of differences was determined by the t-test or Mann-Whitney U test. n.s.: not significant, *P< 0.05. Data are from at least two independent experiments. Supplementary Fig. 2. Induction of NK dCIS from two independent volunteers. (Left and right) NK dCIS data from Volunteer 1 and 2, respectively. (Top) Graph shows the NKC expansion ratio 3, 5, and 7 days after electroporation. Data are the mean ± SD (n = 5). The significance of differences was determined by one-way ANOVA followed by Tukey’s test. n.s.: not significant, **P <0.01, *P < 0.05. (Middle and bottom) Growth inhibition assays of NK mock and NK dCIS on T98G (middle) and U251MG cells (bottom). E:T ratios were 0.5:1 and 1:1 (0.5 × 106:1 × 10^6^ and 1 × 106:1 × 10^6^), respectively. Red, pink, green, light blue, and dark blue lines indicate GBM cells only (E:T = 0:1), NK mock (E:T = 0.5:1), NK dCIS (E:T = 0.5:1) NK mock (E:T = 1:1), and NK dCIS (E:T = 1:1), respectively. Data are the mean ± SD (n = 3–4). Supplementary Fig. 3. CIS mRNA expression in the CIS protein-deleted NKCs. Graph shows mRNA expression in the CIS protein-deleted NKCs extracted from microarray data. Blue and red graphs denote NK mock and NK dCIS, respectively. Data are the mean ± SD (n = 3). The significance of differences was determined by the t-test. *P< 0.05. Supplementary Fig. 4. The influence of CIS expression and OT/OF effects in NK dCIS 14 da [file 13046_2023_2770_MOESM3_ESM.zip › supple fig 1.PNG]

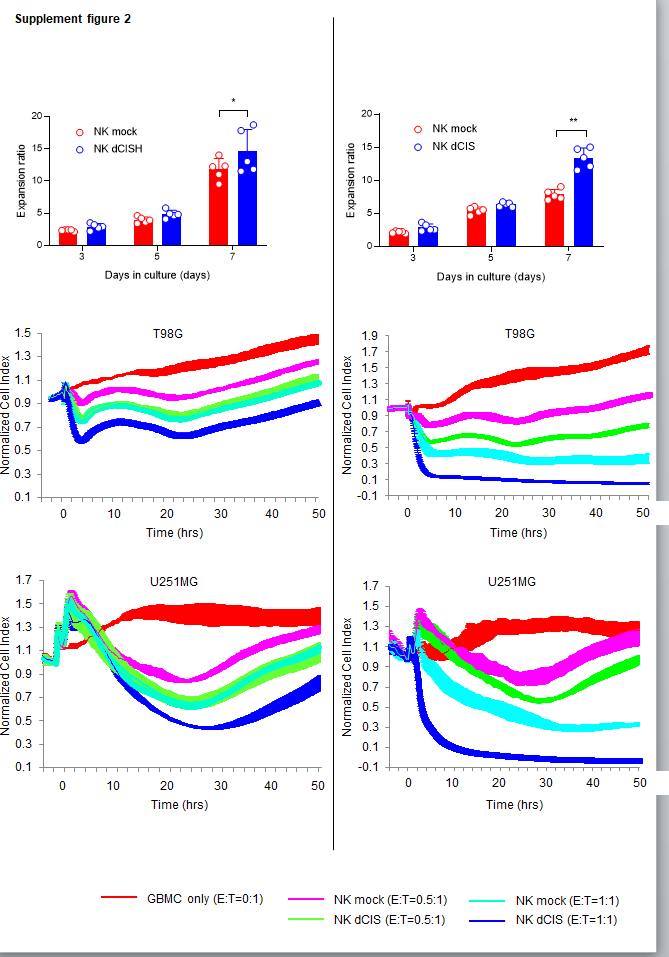

Supplement: Supplementary file 3 — Additional file 3: Supplementary Fig. 1. Effect of CIS deletion on NKC receptor expression on the expanded NKCs. a Representative histogram of NK mock and NK dCIS analyzed by flow cytometry for NK activating and inhibitory receptors. Seven representative activating and inhibitory receptors are shown. The histograms were gated by the CD56-positive fraction. Blue, red, and gray histograms represent NK dCIS, NK mock, and negative background (NB) cells, respectively. b Graph depicts normalized MFI. Blue and red bars indicate NK mock and NK dCIS, respectively. Data are the mean ± SD, n = 4. The significance of differences was determined by the t-test or Mann-Whitney U test. n.s.: not significant, *P< 0.05. Data are from at least two independent experiments. Supplementary Fig. 2. Induction of NK dCIS from two independent volunteers. (Left and right) NK dCIS data from Volunteer 1 and 2, respectively. (Top) Graph shows the NKC expansion ratio 3, 5, and 7 days after electroporation. Data are the mean ± SD (n = 5). The significance of differences was determined by one-way ANOVA followed by Tukey’s test. n.s.: not significant, **P <0.01, *P < 0.05. (Middle and bottom) Growth inhibition assays of NK mock and NK dCIS on T98G (middle) and U251MG cells (bottom). E:T ratios were 0.5:1 and 1:1 (0.5 × 106:1 × 10^6^ and 1 × 106:1 × 10^6^), respectively. Red, pink, green, light blue, and dark blue lines indicate GBM cells only (E:T = 0:1), NK mock (E:T = 0.5:1), NK dCIS (E:T = 0.5:1) NK mock (E:T = 1:1), and NK dCIS (E:T = 1:1), respectively. Data are the mean ± SD (n = 3–4). Supplementary Fig. 3. CIS mRNA expression in the CIS protein-deleted NKCs. Graph shows mRNA expression in the CIS protein-deleted NKCs extracted from microarray data. Blue and red graphs denote NK mock and NK dCIS, respectively. Data are the mean ± SD (n = 3). The significance of differences was determined by the t-test. *P< 0.05. Supplementary Fig. 4. The influence of CIS expression and OT/OF effects in NK dCIS 14 da [file 13046_2023_2770_MOESM3_ESM.zip › supple fig 2.PNG]

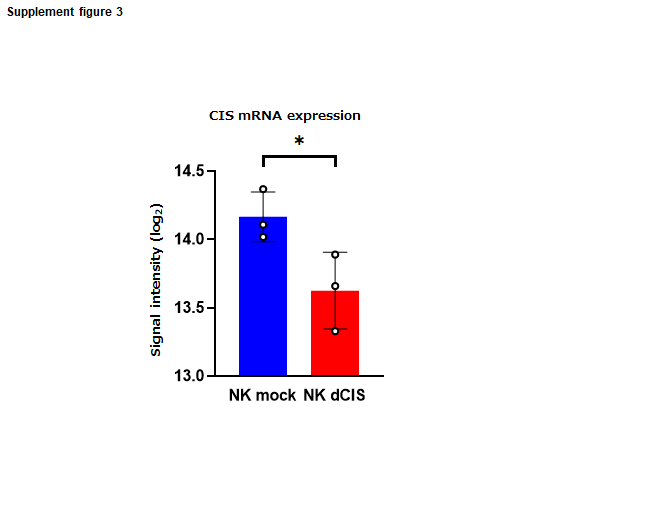

Supplement: Supplementary file 3 — Additional file 3: Supplementary Fig. 1. Effect of CIS deletion on NKC receptor expression on the expanded NKCs. a Representative histogram of NK mock and NK dCIS analyzed by flow cytometry for NK activating and inhibitory receptors. Seven representative activating and inhibitory receptors are shown. The histograms were gated by the CD56-positive fraction. Blue, red, and gray histograms represent NK dCIS, NK mock, and negative background (NB) cells, respectively. b Graph depicts normalized MFI. Blue and red bars indicate NK mock and NK dCIS, respectively. Data are the mean ± SD, n = 4. The significance of differences was determined by the t-test or Mann-Whitney U test. n.s.: not significant, *P< 0.05. Data are from at least two independent experiments. Supplementary Fig. 2. Induction of NK dCIS from two independent volunteers. (Left and right) NK dCIS data from Volunteer 1 and 2, respectively. (Top) Graph shows the NKC expansion ratio 3, 5, and 7 days after electroporation. Data are the mean ± SD (n = 5). The significance of differences was determined by one-way ANOVA followed by Tukey’s test. n.s.: not significant, **P <0.01, *P < 0.05. (Middle and bottom) Growth inhibition assays of NK mock and NK dCIS on T98G (middle) and U251MG cells (bottom). E:T ratios were 0.5:1 and 1:1 (0.5 × 106:1 × 10^6^ and 1 × 106:1 × 10^6^), respectively. Red, pink, green, light blue, and dark blue lines indicate GBM cells only (E:T = 0:1), NK mock (E:T = 0.5:1), NK dCIS (E:T = 0.5:1) NK mock (E:T = 1:1), and NK dCIS (E:T = 1:1), respectively. Data are the mean ± SD (n = 3–4). Supplementary Fig. 3. CIS mRNA expression in the CIS protein-deleted NKCs. Graph shows mRNA expression in the CIS protein-deleted NKCs extracted from microarray data. Blue and red graphs denote NK mock and NK dCIS, respectively. Data are the mean ± SD (n = 3). The significance of differences was determined by the t-test. *P< 0.05. Supplementary Fig. 4. The influence of CIS expression and OT/OF effects in NK dCIS 14 da [file 13046_2023_2770_MOESM3_ESM.zip › supple fig 3.PNG]

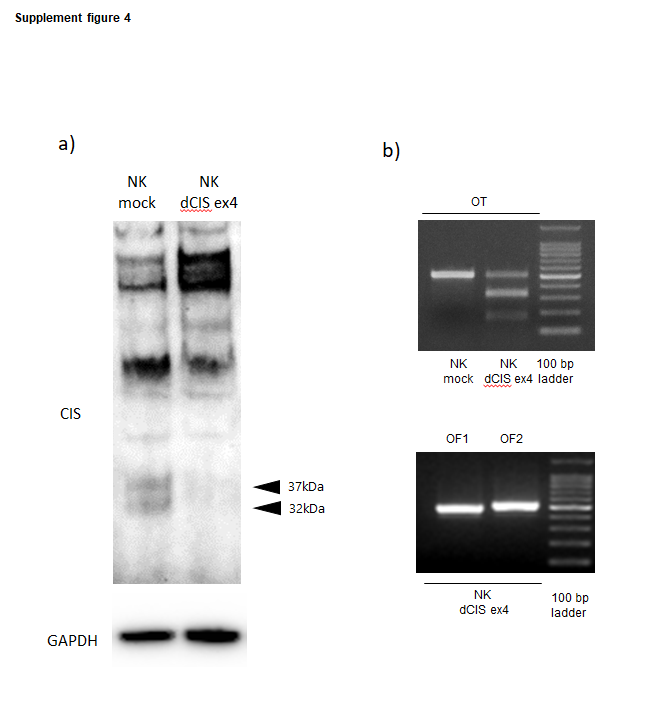

Supplement: Supplementary file 3 — Additional file 3: Supplementary Fig. 1. Effect of CIS deletion on NKC receptor expression on the expanded NKCs. a Representative histogram of NK mock and NK dCIS analyzed by flow cytometry for NK activating and inhibitory receptors. Seven representative activating and inhibitory receptors are shown. The histograms were gated by the CD56-positive fraction. Blue, red, and gray histograms represent NK dCIS, NK mock, and negative background (NB) cells, respectively. b Graph depicts normalized MFI. Blue and red bars indicate NK mock and NK dCIS, respectively. Data are the mean ± SD, n = 4. The significance of differences was determined by the t-test or Mann-Whitney U test. n.s.: not significant, *P< 0.05. Data are from at least two independent experiments. Supplementary Fig. 2. Induction of NK dCIS from two independent volunteers. (Left and right) NK dCIS data from Volunteer 1 and 2, respectively. (Top) Graph shows the NKC expansion ratio 3, 5, and 7 days after electroporation. Data are the mean ± SD (n = 5). The significance of differences was determined by one-way ANOVA followed by Tukey’s test. n.s.: not significant, **P <0.01, *P < 0.05. (Middle and bottom) Growth inhibition assays of NK mock and NK dCIS on T98G (middle) and U251MG cells (bottom). E:T ratios were 0.5:1 and 1:1 (0.5 × 106:1 × 10^6^ and 1 × 106:1 × 10^6^), respectively. Red, pink, green, light blue, and dark blue lines indicate GBM cells only (E:T = 0:1), NK mock (E:T = 0.5:1), NK dCIS (E:T = 0.5:1) NK mock (E:T = 1:1), and NK dCIS (E:T = 1:1), respectively. Data are the mean ± SD (n = 3–4). Supplementary Fig. 3. CIS mRNA expression in the CIS protein-deleted NKCs. Graph shows mRNA expression in the CIS protein-deleted NKCs extracted from microarray data. Blue and red graphs denote NK mock and NK dCIS, respectively. Data are the mean ± SD (n = 3). The significance of differences was determined by the t-test. *P< 0.05. Supplementary Fig. 4. The influence of CIS expression and OT/OF effects in NK dCIS 14 da [file 13046_2023_2770_MOESM3_ESM.zip › supple fig 4.PNG]

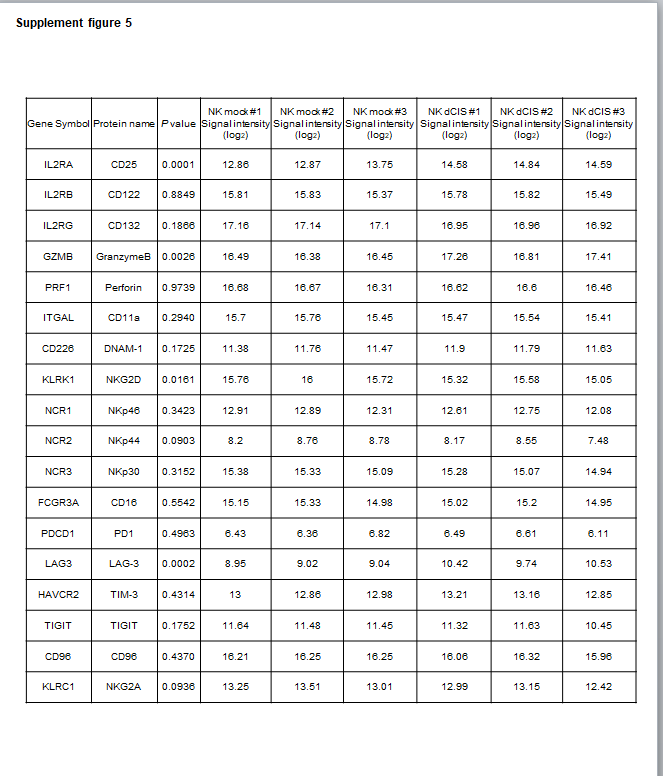

Supplement: Supplementary file 3 — Additional file 3: Supplementary Fig. 1. Effect of CIS deletion on NKC receptor expression on the expanded NKCs. a Representative histogram of NK mock and NK dCIS analyzed by flow cytometry for NK activating and inhibitory receptors. Seven representative activating and inhibitory receptors are shown. The histograms were gated by the CD56-positive fraction. Blue, red, and gray histograms represent NK dCIS, NK mock, and negative background (NB) cells, respectively. b Graph depicts normalized MFI. Blue and red bars indicate NK mock and NK dCIS, respectively. Data are the mean ± SD, n = 4. The significance of differences was determined by the t-test or Mann-Whitney U test. n.s.: not significant, *P< 0.05. Data are from at least two independent experiments. Supplementary Fig. 2. Induction of NK dCIS from two independent volunteers. (Left and right) NK dCIS data from Volunteer 1 and 2, respectively. (Top) Graph shows the NKC expansion ratio 3, 5, and 7 days after electroporation. Data are the mean ± SD (n = 5). The significance of differences was determined by one-way ANOVA followed by Tukey’s test. n.s.: not significant, **P <0.01, *P < 0.05. (Middle and bottom) Growth inhibition assays of NK mock and NK dCIS on T98G (middle) and U251MG cells (bottom). E:T ratios were 0.5:1 and 1:1 (0.5 × 106:1 × 10^6^ and 1 × 106:1 × 10^6^), respectively. Red, pink, green, light blue, and dark blue lines indicate GBM cells only (E:T = 0:1), NK mock (E:T = 0.5:1), NK dCIS (E:T = 0.5:1) NK mock (E:T = 1:1), and NK dCIS (E:T = 1:1), respectively. Data are the mean ± SD (n = 3–4). Supplementary Fig. 3. CIS mRNA expression in the CIS protein-deleted NKCs. Graph shows mRNA expression in the CIS protein-deleted NKCs extracted from microarray data. Blue and red graphs denote NK mock and NK dCIS, respectively. Data are the mean ± SD (n = 3). The significance of differences was determined by the t-test. *P< 0.05. Supplementary Fig. 4. The influence of CIS expression and OT/OF effects in NK dCIS 14 da [file 13046_2023_2770_MOESM3_ESM.zip › supple fig 5.PNG]
